# Supplementary material for: Astronomical aspects of Group E-type complexes and implications for understanding ancient Maya architecture and urban planning
Source: PLoS One. 2021 Apr 27;16(4):e0250785. doi: 10.1371/journal.pone.0250785 (PMC8078784; doi:10.1371/journal.pone.0250785)
Supplement: S1 Text — (DOCX) [file pone.0250785.s001.docx]

# S1 Text: Details on materials and methods

The alignment data for most E Groups and other structures included in this study (S1 Table) are based on field measurements. In several cases they were obtained from lidar imagery in UTM projection; cartographic azimuths were corrected for grid convergence to true (astronomical) azimuths.

Field measurements were carried out with a Suunto Tandem compass, which allows angles to be read with the precision of ±0.25°, with a TruPulse 360 ​​device, in which an electronic compass and a distanciometer are integrated, or with a theodolite and astronomical reference (the Sun), employing standard archaeoastronomical techniques and computing procedures [1-5]. Magnetic azimuths were corrected to true (astronomical) azimuths for the values of local magnetic declination,​​ obtained for each site using the National Oceanic and Atmospheric Administration (NOAA) calculator, available online (<https://www.ngdc.noaa.gov/geomag/calculators/magcalc.shtml>). The validity of the predictive model used by this calculator was often verified by field measurements: along various lines measured with theodolite and astronomical reference, magnetic readings were also taken. For the purposes of analyses (see below), each azimuth was assigned an error estimated on the basis of the present state of the building and the resulting uncertainties regarding the originally intended value. The geographical coordinates of each site, necessary in calculations, were determined with GPS devices.

In a number of E Groups, the alignments cannot be measured directly, because the vegetation prevents the view from the western pyramid to the relevant points of the eastern platform. However, since most plazas are free of vegetation, it was possible to find spots from where the relevant points on the structures could be sighted. Two methods were used in these measurements, one with the TruPulse 360 ​​device and the other with theodolite and astronomical fix. With the TruPulse 360 ​​device it is possible, by sighting from the same point, to take readings of two different points and the device calculates the azimuth of the line connecting them. Employing the other method, it was necessary to take reading from two points. The situation is presented in Figure S1: points B and D correspond to the observation points (stations for the theodolite), while letters A and C designate the endpoints of the line for which the azimuth needs to be calculated. From each station the horizontal angles towards the three other points were measured; by taking readings of the Sun from at least one point, it was possible to calculate the astronomical azimuths of all the lines. To calculate the angle designated as *α_1_* in Figure S1, necessary for calculating the azimuth of the line AC, I derived the following formula, using trigonometric expressions:

$$\tan\alpha_{1}=\frac{\sin\alpha}{\cos\alpha+\frac{\sin\beta_{2}\sin\delta}{\sin\beta\sin\delta_{2}}}$$

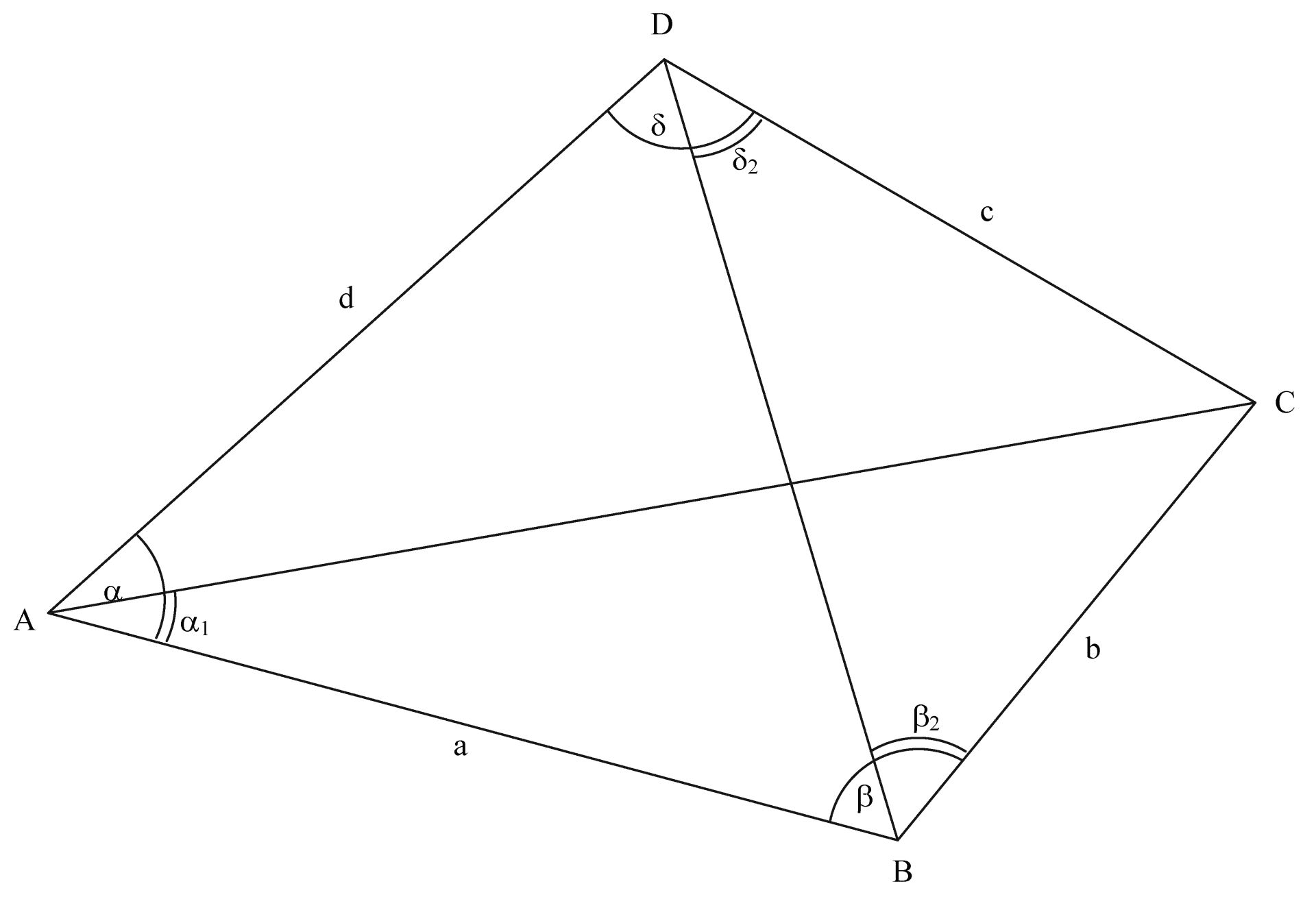


Figure S1. Illustration of the method for calculating the azimuth of the line AC by taking readings with a theodolite and astronomical reference from points B and D.

While an alignment is defined by its azimuth (angle in the horizontal plane, measured clockwise from the north), any potential astronomical target of an alignment can be identified only by determining the declination of the corresponding horizon point. The declination, a celestial coordinate that expresses angular distance measured from the celestial equator to the north and south, depends on the azimuth of the alignment, geographic latitude of the observer, and the horizon altitude corrected for atmospheric refraction.^[[1]](#footnote-1)^ If it was impossible to measure horizon altitudes in the field, due to visibility problems, they were determined with the aid of 3-arcsecond SRTM data (<http://srtm.csi.cgiar.org/srtmdata/>), employing Horizon 0.12a software developed by A. G. K. Smith (<http://www.agksmith.net/horizon/>). Since the SRTM relief model is not entirely accurate, the calculated horizon altitudes may have errors, which in most cases are insignificant, but may be considerable if the horizon line is within a short distance. The errors assigned to declinations (S1 Table) are based on the errors estimated for both azimuths and horizon altitudes.

As demonstrated by previous research, including statistical analyses, the orientations in Mesoamerican civic and ceremonial architecture refer predominantly to astronomical phenomena observable on the horizon, i.e. to the rising and setting points of the heavenly bodies [1,4,5,8-11]. Therefore, all data analyzed (S1 Table) refer to the events observable on the natural horizon. Even for E Groups where a building currently blocks the view to the natural horizon, it is possible to assume that, at an earlier construction stage, the natural horizon was visible.

The declinations within the solar span (from ca. -23.5° to ca. 23.5°) were converted to Gregorian dates and the intervening intervals were also calculated. The declination of the Sun varies continuously along the tropical year, reaching its extreme values at the solstices (around December 22 and June 22). All other values are attained twice a year, once in each half delimited by the solstices, which means that two sunrise and two sunset dates correspond to each alignment and each date pair divides the year into two intervals, whose sum is equal to the length of the tropical year (currently about 365.2422 days).

The dates determined for declinations within the solar span are given in the proleptic Gregorian calendar, which is the closest approximation to the tropical year, and are valid for the period of construction of the building or E Group in question (S1 Table). Due to secular variations affecting the obliquity of the ecliptic, the length of the tropical year and the heliocentric longitude of the perihelion of the Earth’s orbit (the latter element determining the length of astronomical seasons), on the one hand, and to the intercalation system used in the Gregorian calendar, on the other, one and the same solar declination does not necessarily correspond in any time span to exactly the same Gregorian date. For different periods (Middle Preclassic, Late Preclassic, Early and Late Classic, Late Postclassic), several ordinary Julian years were chosen for computations (450 and 50 BCE, and 350, 699 and 1399 CE, respectively). For each of these years (minor uncertainties regarding the dating of structures have no major relevance, because the dates corresponding to certain solar declinations remain the same for about two or three centuries), solar ephemeris data were generated, employing Horizons Web-Interface provided by the Solar System Dynamics Group of the NASA Jet Propulsion Laboratory (<https://ssd.jpl.nasa.gov/?horizons>). A list containing the Sun’s apparent geocentric declinations calculated for the whole year at intervals of 6 minutes, as well as the corresponding Julian dates and hours, was downloaded and imported to an Excel table. In order to obtain comparable dates, the moment nearest to the vernal equinox (when the Sun’s declination was nearest to 0°) was in all cases taken to be March 21.0, Gregorian (March 21, at 0:00 hours of Universal Time) and all other Julian dates and hours in the table were corrected accordingly.^[[2]](#footnote-2)^ Then the differences between all the declinations listed in the table and the declination corresponding to a particular alignment were calculated; after finding the smallest two differences, which indicated the two Gregorian dates matching the alignment’s declination, the intervening intervals were calculated. One of the two intervals was the exact difference between the two dates, but since one table of ephemeris data comprised only one year, the complementary interval was calculated by subtracting the other from the length of the tropical year, calculated for the year in question with the algorithm given by Meeus and Savoie [12]. As the same procedure had to be repeated for all declinations targeted by the alignments included in the study, a macro routine in Excel was created for these computations. In this routine, the errors of dates and intervals, based on the estimated errors of declinations, were also calculated. All these data are listed in S1 Table, in which the errors of dates are not included, but are implicit in the errors calculated for the intervals.

The attempt to achieve the precision in determining dates and intervals might appear an exaggeration, considering that, due to the current state of the structures, the azimuths of many alignments cannot be accurately determined. However, this effort seemed preferable, so as not to increase the errors that are inevitable.

A rather common procedure for testing for intentionality in astronomical orientations consists of selecting putative astronomical targets, matching them with archaeological alignments, and then conducting some sort of statistical test of the likelihood of coincidence [3,13]. However, as in some previous studies, I have adopted a more objective approach, in which no astronomical target is given a preconceived significance. To analyze the distributions of declinations, dates and intervals, I applied the method known as kernel density estimation (KDE; see [11] for details), in which each value is represented as a Gausssian curve (kernel) with the estimated error considered to represent standard deviation from the nominal value. To produce the distribution function, the kernels representing each data set were then summed up, employing Kernel.xla 1.0e software (developed by S. Ellison, Royal Society of Chemistry: <https://www.rsc.org/Membership/Networking/InterestGroups/Analytical/AMC/Software/>). The advantage of this method over histograms is in that the errors assigned to similar values tend to cancel out; it can thus be expected that the most prominent peaks of the resulting curves, which present relative frequency distributions, closely correspond to the values targeted by particular orientation groups.

# References

1. Aveni AF. Skywatchers: A revised and updated version of Skywatchers of ancient Mexico. Austin: University of Texas Press; 2001.
2. Hawkins GS. Astro-archaeology. Vistas in Astronomy. 1968; 10: 45–88.
3. Ruggles C. Astronomy in prehistoric Britain and Ireland. New Haven - London: Yale University Press; 1999.
4. Šprajc I. Orientaciones astronómicas en la arquitectura prehispánica del centro de México. México: Instituto Nacional de Antropologí­a e Historia; 2001 (Colección Científica 427).
5. Šprajc I, Sánchez Nava PF. Orientaciones astronómicas en la arquitectura de Mesoamérica: Oaxaca y el Golfo de México. Ljubljana: Založba ZRC; 2015 (Prostor, kraj, čas 8). <https://doi.org/10.3986/9789612548162>
6. Bennett GG. The calculation of astronomical refraction in marine navigation. The Journal of Navigation 1982; 35(2): 255–259.
7. Young AT. Sunset science. IV: low-altitude refraction. The Astronomical Journal 2004: 127(6): 3622-3637.
8. Aveni A, Hartung H. Maya city planning and the calendar. Philadelphia; 1986. (Transactions of the American Philosophical Society 76, Part 7).
9. Sánchez Nava PF, Šprajc I. Orientaciones astronómicas en la arquitectura maya de las tierras bajas. México: Instituto Nacional de Antropología e Historia; 2015 (Colección Arqueología, Serie Logos).
10. Tichy F. Die geordnete Welt indianischer Völker. Stuttgart: Franz Steiner Verlag; 1991 (Das Mexiko-Projekt der Deutschen Forschungsgemeinschaft 21).
11. González-García AC, Šprajc I. Astronomical significance of architectural orientations in the Maya Lowlands: a statistical approach. Journal of Archaeological Science: Reports 2016; 9: 191-202. <https://doi.org/10.1016/j.jasrep.2016.07.020>
12. Meeus J, Savoie D. The history of the tropical year. Journal of the British Astronomical Association 1992; 102(1): 40–42.
13. Aveni, AF. Evidence and intentionality: On method in archaeoastronomy. In: Bostwick, TW, Bates B, editors. Viewing the sky through past and present cultures: Selected papers from the Oxford VII International Conference on Archaeoastronomy. Phoenix: City of Phoenix Parks and Recreation Department; 2006, p. 57-70 (Pueblo Grande Museum Anthropological Papers No. 15).

1. The formula is:

   *δ* = arc sin (sin *φ* sin *h* + cos *φ* cos *h* cos *A*) (1)

   where *δ* is the declination, *φ* is the geographic latitude, *A* is the azimuth and *h* is the altitude of the observed point corrected for atmospheric refraction:

   *h* = *H* – *r* (2)

   where *H* is the observed altitude and *r* is the refraction angle. For calculating *r*, refraction factor *R* has to be calculated. I employed formula 3, in which the altitude *H* is expressed in decimal degrees and the result *R* is in arc minutes (formula G in [6]):

   *R =* cot [*H* + 7.31/(*H* + 4.4)] (3)

   The exact refraction values depend not only on the altitude *H* of the sighted point but also on concrete atmoshperic conditions (temperature, air pressure); at altitudes near the mathematical horizon (horizontal plane) they exhibit unpredictable variations [7]. To correct the refraction factor *R* for the height above sea level (*hasl*) of the observing point, I employed Hawkins’ formula 4 [2] and obtained the value of *r*, sufficiently accurate for our purposes:

   *r = R* × e*^–asnm^*^/8400^ (4)

   The value of *r* was then employed in formula 2, resulting in the value of *h*, necessary for calculating the declination with formula 1. [↑](#footnote-ref-1)
2. If the true moment of the equinox in each year used for determining the dates had been considered, the analysis of their distribution would yield unreliable results, since the dates corresponding to the same declination would present variations of up to about ±1 day. Put more precisely, by correlating the vernal equinox invariably with the same date, the variations resulting from the intercalation system in the Gregorian calendar are eliminated, but those due to the aforementioned secular variations in orbital elements remain. That is why, in some cases, the pair of dates corresponding to the same declination changed along the time span covered by the study. [↑](#footnote-ref-2)
