# Supplementary material for: Astronomical aspects of Group E-type complexes and implications for understanding ancient Maya architecture and urban planning
Source: PLoS One. 2021 Apr 27;16(4):e0250785. doi: 10.1371/journal.pone.0250785 (PMC8078784; doi:10.1371/journal.pone.0250785)
Supplement: Attachment — Submitted filename: Response to Reviewers.docx [file pone.0250785.s003.docx]

Dear Dr. Freeman,

I wish to give my thanks to you and the reviewers for all the comments and for giving me the opportunity to revise my paper. Below are my responses indicating the modified sections of the revised manuscript and the corresponding line numbers, and explaining the reasons for which I believe several objections are unwarranted.

Please note that, due to the known issue in MS Word, line numbering in my revised manuscript in which all changes have been accepted does not agree with that in the Revised Manuscript with Track Changes; in my replies below I always refer to line numbers in the latter version, to make my modifications clearly noticeable.

Sincerely,

Ivan Šprajc

PONE-D-20-26825
Astronomical aspects of Group E-type complexes and implications for understanding ancient Maya architecture and urban planning
PLOS ONE

Dear Dr. Šprajc,

Thank you for submitting your manuscript to PLOS ONE. After careful consideration, we feel that it has merit but does not fully meet PLOS ONE’s publication criteria as it currently stands. Therefore, we invite you to submit a revised version of the manuscript that addresses the points raised during the review process.

Your paper has been reviewed by two well accomplished scholars of Mesoamerican archaeology, including one with some expertise in archaeoastronomy. Reviewer 1 believes that the paper meets Plos One’s standards of scientific soundness. Reviewer 2 believes that the paper does not meet Plos One’s standards of scientific soundness.  Reviewer 2 raises important critiques that must be adequately addressed before the paper can meet Plos One’s publication criteria. I list the three most important points below (though all critiques from both reviewers are useful).

1. ``The author has not demonstrated that the observed observations are not due to chance. There is some clustering of alignments with the (rather large) range of predicted values, but many alignments are far from a prediction.”  This bears directly on the criterion of appropriate and rigorous statistical analysis. In addition to R2’s critique, from my own reading, the justification of the use of KDEs needs more development.

I have modified the text in some parts of Introduction, Materials and methods, and supplementary S1 Text, hopefully making clearer the nature and utility of KDE analyses. I address these and other issues more thoroughly below in the responses to Reviewer 2.

2. ``Most of the exercise seems composed of post-hoc reasoning, without testing of hypotheses. How would one know if the author is wrong? No empirical means of choosing between a correct and an incorrect interpretation are given.” This bears directly on the issue of whether the data support the conclusions of the paper.

Please see my replies to Reviewer 2, below.

3. More evidence is needed to justify the assertion that calendars were used to ``facilitate an efficient scheduling of agricultural works.”

Please see my replies to Reviewer 2, below.

Please submit your revised manuscript by Jan 04 2021 11:59PM. If you will need more time than this to complete your revisions, please reply to this message or contact the journal office at [plosone@plos.org](mailto:plosone@plos.org). When you're ready to submit your revision, log on to <https://www.editorialmanager.com/pone/> and select the 'Submissions Needing Revision' folder to locate your manuscript file.

Please include the following items when submitting your revised manuscript:

- A rebuttal letter that responds to each point raised by the academic editor and reviewer(s). You should upload this letter as a separate file labeled 'Response to Reviewers'.
- A marked-up copy of your manuscript that highlights changes made to the original version. You should upload this as a separate file labeled 'Revised Manuscript with Track Changes'.
- An unmarked version of your revised paper without tracked changes. You should upload this as a separate file labeled 'Manuscript'.

If you would like to make changes to your financial disclosure, please include your updated statement in your cover letter. Guidelines for resubmitting your figure files are available below the reviewer comments at the end of this letter.

If applicable, we recommend that you deposit your laboratory protocols in protocols.io to enhance the reproducibility of your results. Protocols.io assigns your protocol its own identifier (DOI) so that it can be cited independently in the future. For instructions see: <http://journals.plos.org/plosone/s/submission-guidelines#loc-laboratory-protocols>

We look forward to receiving your revised manuscript.

Kind regards,

Jacob Freeman
Academic Editor
PLOS ONE

Journal Requirements:

When submitting your revision, we need you to address these additional requirements.

 1. Please ensure that your manuscript meets PLOS ONE's style requirements, including those for file naming. The PLOS ONE style templates can be found at

<https://journals.plos.org/plosone/s/file?id=wjVg/PLOSOne_formatting_sample_main_body.pdf> and

<https://journals.plos.org/plosone/s/file?id=ba62/PLOSOne_formatting_sample_title_authors_affiliations.pdf>

2. In your manuscript, please provide additional information regarding the specimens used in your study. Ensure that you have reported specimen numbers and complete repository information, including museum name and geographic location.

If permits were required, please ensure that you have provided details for all permits that were obtained, including the full name of the issuing authority, and add the following statement:

'All necessary permits were obtained for the described study, which complied with all relevant regulations.'

If no permits were required, please include the following statement:

'No permits were required for the described study, which complied with all relevant regulations.'

For more information on PLOS ONE's requirements for paleontology and archaeology research, see <https://journals.plos.org/plosone/s/submission-guidelines#loc-paleontology-and-archaeology-research>.

[Note: HTML markup is below. Please do not edit.]

Reviewers' comments:

Reviewer's Responses to Questions

**Comments to the Author**

1. Is the manuscript technically sound, and do the data support the conclusions?

The manuscript must describe a technically sound piece of scientific research with data that supports the conclusions. Experiments must have been conducted rigorously, with appropriate controls, replication, and sample sizes. The conclusions must be drawn appropriately based on the data presented.

Reviewer #1: Yes

Reviewer #2: Partly

2. Has the statistical analysis been performed appropriately and rigorously?

Reviewer #1: Yes

Reviewer #2: No

3. Have the authors made all data underlying the findings in their manuscript fully available?

The [PLOS Data policy](http://www.plosone.org/static/policies.action#sharing) requires authors to make all data underlying the findings described in their manuscript fully available without restriction, with rare exception (please refer to the Data Availability Statement in the manuscript PDF file). The data should be provided as part of the manuscript or its supporting information, or deposited to a public repository. For example, in addition to summary statistics, the data points behind means, medians and variance measures should be available. If there are restrictions on publicly sharing data—e.g. participant privacy or use of data from a third party—those must be specified.

Reviewer #1: Yes

Reviewer #2: Yes

4. Is the manuscript presented in an intelligible fashion and written in standard English?

PLOS ONE does not copyedit accepted manuscripts, so the language in submitted articles must be clear, correct, and unambiguous. Any typographical or grammatical errors should be corrected at revision, so please note any specific errors here.

Reviewer #1: Yes

Reviewer #2: Yes

5. Review Comments to the Author

Please use the space provided to explain your answers to the questions above. You may also include additional comments for the author, including concerns about dual publication, research ethics, or publication ethics. (Please upload your review as an attachment if it exceeds 20,000 characters)

Reviewer #1: This is a well-executed and well-written paper that deserves to be published. Sprajic has presented a solid contribution in archaeoastronomy in which he reanalyzes the function of E Groups, an architectural complex that forms the earliest public architecture at most ancient Maya sites. Whereas they were originally conceived to have reflected the passage of the sun in terms of solstices and equinoxes, Sprajic demonstrates that this cannot be the case and instead argues that they only share common east-west alignments that could have been useful in the past in breaking up the solar year into smaller units that were likely related to agricultural processes. His command of archaeoastronomy goes beyond most of his peers and he has done much to redeem this form of research by explicitly documenting his research processes. For anyone interested in the origins of Maya, and Mesoamerican, civilization, this article will be extremely useful.
detailed comments:
Line 75: besides “principles of Maya architectural and urban planning,” these alignments could be representative of Maya cosmological systems

Corrected (lines 75-76).

Line 179: rather than “its,” the word should probably be “their”

Formulation modified (line 201).

Line 225: since Ceiba is part of Caracol in the Late Classic Period, perhaps “locale” is better than “site”

Corrected (line 251).

Line 325: need to clarify that lateral alignments does not refer to deposits in lateral buildings, which do occur at Uaxactun, Caracol, and Tikal during the Early Classic era

Clarification added (lines 374-377).

Line 371: should also cite reference [5] for the Early Classic date of this western pyramid

Added (line 424).

Line 504: please explain what this shift did

Explained (lines 561-564).

Line 648: add a reference for construction largely ceasing in the Early Classic Period (perhaps reference [6])

Added (line 765).

Line 669: the lateral buildings sometimes hold later deposits, but do not reflect the original alignments

Clarification added (lines 792-793).

Line 720: suggest adding new reference after “through many centuries”:

Chase, DZ, McAnany, PA, Sabloff, JA. Epilogue: E Groups and their Significance to the Ancient Maya. In: Freidel, DA, Chase, AF, Dowd, AS, Murdock, J, editors. Maya E Groups: Calendars, astronomy, and urbanism in the early lowlands. Gainesville: University Press of Florida; 2017, p. 578-582.

Reference added (lines 858 & 1259-1261).

Reviewer #2: I find much that is problematic in this paper, but I am not sufficiently versed in the methods and theory of astronomy or archaeoastronomy to present a proper critique. The following are among the negative features that occur to me.

1. The author has not demonstrated that the observed observations are not due to chance. There is some clustering of alignments with the (rather large) range of predicted values, but many alignments are far from a prediction.

In the case of lateral alignments in E Groups, it is precisely because of the lack of their patterned distribution that I reject some previous hypotheses about their astronomical significance. Regarding the central axes of E Groups and orientations of other buildings, I show that there are several likely targets. I am not sure what is meant by predictions; as I have now made explicit in S1 Text, the KDE method does not test intentionality of some predicted values, selected on the basis of their preconceived significance, but rather suggests the values in a data set that are unlikely to be coincidental. If not every alignment can be explained, this is so not only because most likely not each and every orientation was astronomically functional (so there is inevitably some noise in their KDE distribution) but also because several values have been assigned relatively large errors estimated on the basis of the current state of preservation of the buildings involved. Due to these errors, some nearby values (represented as Gaussian kernels) blend when they are summed up, resulting in no significant peak (see lines 256-258, 317-318, 817-820). This is simply an inherent characteristic of the method, making impossible to discern certain possibly intended values, but it does not invalidate it. My interpretations of data distribution are limited to some prominent peaks, which are clearly visible and can hardly be attributed to chance. I realize that different statistical analyses could be performed, but these would require a separate and much more technical paper, comparable to the one we published before and in which a more comprehensive and rigorous statistical exercise was based on a much larger data set from the Maya Lowlands (ref. 25). I am also perfectly aware that not each and every conclusion I propose in this study is rigorously demonstrable, but my interpretations are consistent with and build on the results of previous research, even if some of them must be regarded as tentative and subject to changes once further data become available.

2. It seems implausible to me that the basic principle of orientation to alignments whose sunrise dates are separated by 20 days, or any of a series of multiples of 20 days. This is a basic claim of this author in many publications over the years, but it does not seem plausible to me. The author has proposed this not only for the Maya, but also for the Aztecs and other Mesoamerican cultures. I would think that if such a practice was indeed pursued in ancient Mesoamerica—particularly in the Aztec case—there would be some clue or hint of it in the copious ethnohistoric literature on the Aztecs. The closest reference is a single documentary account that the Aztec main temple was aligned with sunrise on the solstice, which does not mention 20-day intervals or multiples thereof.

In a succinct way I would say that absence of evidence is not evidence of absence. In general, if we only had to rely on written evidence, archaeology would not be able to add anything to our knowledge of the past. More specifically, the absence of written reports does not preclude the possibility that astronomical alignments with certain characteristics existed, particularly because relevant evidence is provided by material remains, as well as by other types of contextual data. I must clarify that the importance of multiples of 20 days in laying out astronomical orientations in Maya and other Mesoamerican public architecture was first noticed by Aveni and Hartung (ref. 14) and Tichy (ref. 17) and later reconfirmed by Aveni (ref. 12) and Aveni et al. (ref. 61); I confess it was unfair to omit these references, which are now added in line 107. My own research has resulted in a much broader corpus of data confirming these proposals, while also revealing the significance of multiples of 13 days. In another study (ref. 25), several more rigorous statistical analyses of a much larger alignment data sample from the Maya Lowlands have shown that the significance of the most prominent dates and intervals is above the 3-sigma level. Furthermore, I have been able to find a substantial amount of ethnographic evidence that attests to the surviving importance of these multiples among some present-day communities, as well as of the dates that were most commonly recorded by orientations and which preserve their agriculturally based ceremonial significance. It comes as no surprise that ethnohistoric literature gives no clue regarding this knowledge, most likely reserved for few specialists. Also in the case of early Christian churches in Europe, written evidence is vague, only mentioning that they should be oriented to the east. Only in recent decades have archaeoastronomical studies in different areas unveiled that their orientations were based on quite specific astronomical and calendrical considerations. True, the only textual reference to the astronomical orientation of a Mesoamerican building is Motolinia’s brief statement about the Templo Mayor of Tenochtitlan, but it was shown long ago (ref. 71) that the orientation of the late stages of this temple is perfectly reconcilable not only with Motolinia but also with the suggested importance of the calendrically significant intervals.

3. Most of the exercise seems composed of post-hoc reasoning, without testing of hypotheses. How would one know if the author is wrong? No empirical means of choosing between a correct and an incorrect interpretation are given.

I have modified parts of Introduction, Materials and Methods, and S1 Text, in the attempt to show that my reasoning is post hoc only to the extent that it discloses variations in comparison with the results of former studies. Under the null hypothesis, assuming that there was no intentionality in laying out orientations, or that other possible orientation motives were involved (climate, topography, defense…), we would have a homogenous or uniform distribution of alignments. However, their evident clustering revealed by a number of previous studies allows us to reject the null hypothesis. Since a few decades ago, after the first systematic studies of Mesoamerican architectural orientations were accomplished, it has been argued that no potential orientation motive other than astronomical can account for the characteristic clustering of alignment data (I have now added this clarification in lines 77-82). As put by Aveni and Hartung (ref. 14: pp. 7-8), “if we find alignments that are confined to a narrow azimuthal range in a sample of buildings spread far apart in space … there can be no conceivable way of actually laying out the chosen direction other than by the use of astronomical bodies at the horizon as reference objects”. Obviously, the data allowing such a conclusion reflect a general trend; nobody excludes the possibility, even likelihood, that other (or no specific) orientation motives were involved in laying out particular civic or ceremonial buildings (let alone domestic structures, which have never been included in such studies). Given that so much research has already been accomplished, I really do not see the need for testing alternative (non-astronomical) hypotheses. The results of analyses presented in this paper are based on a distinct data set; they largely agree with those obtained previously and derived from larger alignment data samples from the Maya Lowlands (and even from other Mesoamerican regions), while also revealing some interesting variations. However, this holds for the central axes of E Groups and orientations of other building types; based on the analyses, I also show that previous astronomical hypotheses related to lateral alignments in E Groups, as well as some other proposals, cannot be sustained.

And again, it may not be superfluous to stress that my interpretations are supported not only by quantitative methods, which alone are not sufficient for explaining the cultural significance of orientations, but also, and to an important extent, by various types of contextual evidence.

4. Many of the author’s claims, including some very unlikely claims, are justified by citations to the author’s own works, with little or no consideration of the work of other scholars. The level of self-citation is remarkably high in this paper. Wild, implausible ideas are justified with self-citation, which only casts doubt on the use of self-citation to justify ideas that seem less problematic.

Self-citations are not there to simply justify my claims and highlight my own work, but rather to direct the reader to publications containing exhaustive discussions of the evidence of multiple types that supports my arguments and which was published by a number of researchers duly cited there. To examine all this evidence here would not only be superfluous but would extend this paper to a monograph. On the other hand, and to my defense, I cannot but say that, after the many years of productive and highly respected research by Anthony Aveni, little serious and systematic work on astronomical orientations in Mesoamerica was carried out by other scholars.

The author expresses the idea that Mesoamerican observational calendars were “intended to facilitate an efficient scheduling of agricultural works” (p. 21, line 479), lists four obscure publications of his own as justification, saying they contain the details (see also p. 31, lines 737-741).

The reasons for labeling these publications as “obscure” are enigmatic to me. The four monographs (cited in line 543) were peer-reviewed and are even accessible online. As mentioned above, they contain not only details but abundant evidence and bibliography, for everyone to check and assess, and were also cited in a review article I published in *Journal of Archaeological Research* (ref. 21).

This is a hold-over from the long-discredited view that Maya priests ruled society, deriving their power form their control of the calendar. Without priests interpreting a calendar, peasants would not know when to plant or harvest. This is simply nonsense. Farmers know when to plant and harvest from environmental cues; there is ample ethnographic confirmation of this from small-scale peasant farming today. I have read many ethnographies that confirm this common-sense viewpoint, and I have talked with many Mesoamerican peasants about how they schedule major activities like planting and harvesting. Calendars have little to do with this, apart perhaps from defining the start of a period when farmers begin following environmental cues more closely. The key variable for timing the planting (in central Mexico, at least) is the onset of the spring rains, and this does not happen on a set date. Calendars were NOT needed for “efficient scheduling of agricultural works.” This claim is repeated—even amplified—on page 31, lines 737-741), where we are told that efficient agriculture “could only be based on astronomical observations.” Again, this is nonsense.

In fact, what is long-discredited is this reviewer’s opinion that farmers know when to plant and harvest from environmental cues. They are certainly familiar with seasonal changes in the nature, but they all schedule their activities with the aid of a calendar, whether they are explicit about it or not. In my publications cited in line 107 (refs. 18, 20, 21, 23, 24), a number of ethnographic cases were examined, which show (as I state in lines 101-104) that modern farmers in the Mesoamerican region use Christian feasts as relevant temporal markers, and in some places they still rely on astronomical observations. In section “Significance of alignments” I cite several examples that illustrate specifically the significance of the dates recorded by alignments analyzed in this study; but to include a lengthy discussion of all pertinent evidence in this paper would be repetitive and distractive to the main argument. Nonetheless, to support my view that seasonal environmental changes are not reliable references, I will here mention a few explicit cases.

Discussing modern agricultural practices in a central Mexican region, Bugé (1987, in Grove, ed., *Ancient Chalcatzingo*, Univ. of Texas Press, p. 418) notes that “farmers are poor predictors of the onset of the rains.” According to Villa Rojas (1978, *Los elegidos de Dios: Etnografía de los mayas de Quintana Roo*, INI, Mexico, pp. 315–316), the Maya farmers of Quintana Roo, Mexico, concerned about burning their fields before the first rains come, seek advice from their scribes, who inform them about the weather in the following months by consulting an almanac printed in Mérida. Among the Quiché and Chortí in Guatemala, calendar experts determine agriculturally important dates by observing positions of the Sun and other celestial bodies (Fought 1972, *Chorti (Mayan) Texts 1*, Univ. of Pennsylvania Press, pp. 386, 435; Girard 1948, *El calendario maya-mexica*, Ed. Stylo, Mexico, pp. 49–59; Tedlock 1991, in Broda et al., eds., *Arqueoastronomía y etnoastronomía en Mesoamérica*, UNAM, Mexico). At Nebaj, also in Guatemala, Lincoln (1945, *An Ethnological Study on the Ixil Indians of the Guatemala Highlands*, Microfilm Collection of Manuscripts on Middle American Cultural Anthropology No. 1, Univ. of Chicago Library) reported the so-called solar observatory, where religious specialists watched the Sun for fixing the planting and harvest seasons.

I am afraid the reviewer has not read carefully my argument, or simply chooses to ignore the evidence. I agree that rainy season or planting “does not happen on a set date”; I acknowledge this quite clearly in lines 554-558. There and elsewhere in the paper I also argue that the dates marked by alignments had a ritual, canonical significance. By contending that calendars or astronomical observations were not necessary for agricultural scheduling, the reviewer disregards the important fact that, aside from regulating practical activities, it was necessary to perform appropriate ceremonies, often ahead of time and with precision. This is again something quite clearly demonstrated by ethnographic evidence, not only from Mesoamerica but also from elsewhere. I have now emphasized this aspect and also summarized what Michael Zeilik reported on ethnoastronomy of the historic Pueblos, where the Sun watcher’s astronomical predictions were expected to be correct within a day, to corroborate the effectiveness of rituals (lines 565-579). In Conclusion I reiterate that the orientations, rather than serving purely utilitarian purposes, marked canonical moments appropriate for performing agriculturally important ceremonies, which were considered indispensable for attaining practical goals, such as abundant crops, and thus had a very “practical” function (lines 880-898). Viewed in this emic context, “efficient” agriculture was obviously related to astronomical observations. By dismissing this relationship as nonsense, the reviewer neglects the compelling contextual evidence given in this paper and in cited publications, as well as a more general fact that the earliest calendrical systems (all based on some sort of astronomical observations) appear independently in different parts of the world, but always when agriculture becomes a prevalent subsistence strategy.

The section on E-groups at Maya sites (starting p. 22) is full of speculation, much of it somewhat wild and unconstrained (lines 520-521, or 531-532). Also, the conclusions are full of speculations.

It would help if the reviewer were more specific in pointing out the statements dismissed as speculative. I can only comment on what in original manuscript was stated in lines 520-521 and 531-532 (now lines 593-599 and 611-616). My hypothesis that 11 axes in a Ceibal cache MAY have alluded to 11 trecenas is no more speculative than the proposal—cited immediately below and published by Inomata and Triadan (ref. 43)—that they may have pointed to the winter solstice sunrise. Also, by mentioning the possibility of early origins of the calendar, I only repeat the hypotheses forwarded by other researchers cited there. Rather than unfounded speculations, these are hypotheses based on some evidence and their role is, as in any scientific pursuit, to stimulate further research.

I don’t think any specialists would agree with the assertion (p. 30, line 703) that “astronomical orientations characterize the vast majority of civic and ceremonial buildings in the Maya area” (with self-citations as justification). I have discussed this issue with a number of Maya archaeologists, and none of them would agree with this assertion. Their view is that the astronomical work of Sprajc and others is a fringe activity that can be ignored for its improbable interpretations. I must admit that I am not aware of published critiques of this assertion. It is repeated on page 31 (lines 732).

My self-citations were admittedly insufficient to support my statement. I have now added other references (line 837), which demonstrate that my work has only extended what was previously recognized by other scholars. If some Maya archaeologists disagree with my assertion, I am wondering on what grounds, because nobody has ever published a rebuttal. I know that quite a number of archaeologists view archaeoastronomy with skepticism, which is likely due to examples of bad scholarship in this relatively new research field (though not uncommon in other scientific disciplines either). But the assertion that my work (by the way, I am an archaeologist) and that of others is a fringe activity is a lamentably aprioristic generalization, revealing the reviewer’s lack of readiness to evaluate the evidence. It is as if someone expressed disbelief in stratigraphy, radiocarbon dating, or ceramic analyses. I would even say that, while doubt could be cast on the trustworthiness of archaeological documentation presented in publications (considering that archaeological record is inevitably lost in the process of excavation and the situation documented or the objectivity in data sampling in many cases cannot be verified), the orientations in architecture are still there for anyone to measure and check again, as well as to reanalyze. As admitted by Anschuetz et al. (2001: 179), “physical alterations of spaces that correspond to astronomical or cosmological phenomena are some of the most testable aspects of ritual use of landscape.” This review article on landscape archaeology was published in *Journal of Archaeological Research* (vol. 9), which has also featured some archaeoastronomical articles (refs. 13, 21); many others have been published in mainstream journals such as *American Antiquity* (ref. 16), *Latin American Antiquity* (refs. 28, 61, 66), *Journal for the History of Astronomy* (refs. 51, 71), *Journal of Archaeological Science* (ref. 25), *Ancient Mesoamerica* (ref. 58), or even *Science* (e.g., Aveni et al., vol. 188, no. 4192, 1975; Ghezzi & Ruggles, vol. 315, no. 5816, 2007). It is hard to believe that all these journals would have published products of some “fringe activity”.

I don’t understand figures 11 and 13.

Regarding Fig. 11, the Sun sets on September 12 along the main axis of Nixtun Ch’ich’, after 20 days it rises along this axis on October 2 and then again after 160 days on March 11, and after 20 days it sets again along the axis on March 31, 200 days after sunset on September 12. Analogous explanation applies to Fig 13. If necessary, I can expand what is already explained in the text (lines 678-679).

6. PLOS authors have the option to publish the peer review history of their article ([what does this mean?](https://journals.plos.org/plosone/s/editorial-and-peer-review-process#loc-peer-review-history)). If published, this will include your full peer review and any attached files.


If you choose “no”, your identity will remain anonymous but your review may still be made public.


**Do you want your identity to be public for this peer review?** For information about this choice, including consent withdrawal, please see our [Privacy Policy](https://www.plos.org/privacy-policy).

Reviewer #1: No

Reviewer #2: No
